# Editorial: Craniomaxillofacial reconstructive and regenerative surgery

**DOI:** 10.3389/fsurg.2026.1857717

**Published:** 2026-05-29

**Authors:** Raphael Capelli Guerra, Jeremie Oliver Piña, Robert Alexander Sader, Bianca Pulino

**Affiliations:** 1 Hospital Sírio-Libanês, Instituto de Ensino e Pesquisa, São Paulo, Brazil; 2 Department of Oral and Maxillofacial Surgery, Faculdade Israelita de Ciências da Saúde Albert Einstein, São Paulo, Brazil; 3 Department of Oral and Maxillofacial Sugery, Einstein Hospital Israelita, São Paulo, Brazil; 4 University of Maryland, Baltimore, MD, United States; 5 Universitätklinikum Frankfurt, Goethe University of Frankfurt, Frankfurt, Germany

**Keywords:** craniomaxillofacial (CMF), innovation, reconstruction (extension) project, regenerative, regenerative surgery:

*Craniomaxillofacial reconstructive and regenerative surgery* continues to evolve as one of the most dynamic and multidisciplinary fields in contemporary surgical science. The complexity of craniofacial defects, whether caused by trauma, oncologic resection, congenital deformity, or aesthetic-functional imbalance, requires not only technical expertise but also a deep integration of anatomy, imaging, biomaterials, microsurgery, and regenerative strategies (1, 2). Increasingly, successful treatment is no longer measured solely by defect closure, but by the restoration of function, esthetics, long-term stability, and reduction of morbidity (3, 4).

This Special Issue was conceived to highlight the breadth of modern craniomaxillofacial surgery, spanning anatomical foundations, trauma care, reconstructive microsurgery, facial aesthetic safety, oncologic reconstruction, and biomaterial science. Collectively, the contributions illustrate how progress in the field is driven by both biological understanding and technical refinement (2, 5).

A first important contribution, Okoń et al. “Morphological classification of the temporalis muscle: anatomical, radiological, and surgical perspectives”, emphasizes the value of anatomical precision in surgical planning. By proposing a morphological framework for the temporalis muscle through anatomical and radiological analysis, this study offers practical insights for surgeons operating in the temporal and craniofacial regions. Such knowledge may improve surgical interpretation, reduce complications, and optimize procedures involving local flaps, temporal access, and aesthetic contour management (6). The work reinforces that anatomy remains the cornerstone of both reconstructive and minimally invasive innovation.

Trauma is represented by Arruda Vasconcelos et al. “Fracture of the frontal sinus: complexity of multidisciplinary treatment”, which highlights the persistent challenges of managing frontal sinus injuries. These fractures often involve not only the osseous framework but also the anterior skull base, nasofrontal outflow tract, orbital structures, and neurosurgical concerns. By addressing the complexity of multidisciplinary treatment, the article underscores the need for coordinated management among maxillofacial surgeons, neurosurgeons, otolaryngologists, and radiologists (7). It reflects a central principle of craniofacial trauma care: optimal outcomes depend on accurate diagnosis, individualized risk assessment, and integrated decision-making (7).

Reconstructive microsurgery is further explored in Soemmer et al. “Tensor fascia lata free flap as salvage solution minimizes donor site morbidity in anterolateral thigh free flap failure—decision making algorithm and a case report”. This article contributes a highly relevant perspective to flap salvage strategies by addressing what can be done when a widely used reconstructive option, the anterolateral thigh flap, is not feasible or fails intraoperatively. The proposed algorithm, coupled with a case report, appears to offer a practical decision-making pathway while prioritizing reduction of donor-site morbidity. Its contribution is especially valuable because reconstructive success often depends not only on the primary plan, but also on the surgeon's ability to respond safely and intelligently when the unexpected occurs (2, 5).

The interface between anatomy and facial aesthetics is addressed in Bravo et al. “Leveraging anatomy to improve safety and efficacy in temple augmentations—a case series of 20 patients”. Temple augmentation has become increasingly relevant in facial harmonization and reconstructive-aesthetic surgery, yet it remains an anatomically sensitive area due to vascular and soft-tissue complexity. This case series appears to reinforce the principle that aesthetic interventions must be anatomy-driven to achieve both safety and reliable outcomes (6). In the broader context of this Special Issue, the article reflects how reconstructive and aesthetic surgery increasingly converge around the same principles: careful anatomical knowledge, technical precision, and complication prevention (6, 7).

Oncologic reconstruction is represented by Ottaviano et al. “Mandibular reconstruction with anterolateral thigh free flap and bridging plate: a retrospective study of 34 oncological cases”. Mandibular defects remain among the most challenging reconstructive problems in head and neck surgery, especially in patients requiring complex ablative procedures (1, 2). This retrospective series contributes clinical evidence on a reconstructive strategy combining soft-tissue free flap transfer with plate-based mandibular restoration. Beyond reporting outcomes in oncologic cases, the article likely contributes to the ongoing debate regarding reconstructive indications, patient selection, functional rehabilitation, and the balance between surgical complexity and clinical benefit (3–5). It highlights the importance of tailoring reconstructive approaches to both disease burden and patient-specific conditions (2, 5).

Finally, biomaterial science is brought into focus in Kauke-Navarro et al. “A systematic review of implant materials for facial reconstructive and aesthetic surgery”. As the field advances, the selection of implant materials has become a central issue in both reconstructive and cosmetic practice. The relevance of this systematic review lies in its potential to synthesize the properties, indications, advantages, and limitations of available implant materials, including considerations related to biocompatibility, stability, infection risk, handling, and esthetic integration (7, 8). This contribution aligns strongly with the regenerative and translational direction of the Special Issue, as biomaterials increasingly shape the future of personalized craniofacial surgery (8).

Taken together, the articles included in this collection reflect the diversity and maturity of *Craniomaxillofacial reconstructive and regenerative surgery*. They remind us that progress in the field emerges from multiple fronts: from anatomical classification to trauma algorithms, from flap salvage to aesthetic safety, from oncologic rehabilitation to evidence-based biomaterial selection (2, 5, 8). Importantly, they also show that innovation is most meaningful when it improves decision-making, enhances safety, and translates into better patient outcomes (3, 4).

We hope this Special Issue will stimulate further dialogue among surgeons, anatomists, radiologists, biomaterial scientists, and multidisciplinary teams involved in craniofacial care. The future of craniomaxillofacial surgery will depend on our capacity to unite precision, biology, and clinical judgment in order to deliver reconstructive solutions that are not only technically successful, but functionally restorative, esthetically sound, and biologically intelligent (4, 8).
